# Knowledge translation of clinical practice guidelines among neurologists: A mixed-methods study

**DOI:** 10.1371/journal.pone.0205280

**Published:** 2018-10-10

**Authors:** Khara M. Sauro, Samuel Wiebe, Jayna Holroyd-Leduc, Carolyn DeCoster, Hude Quan, Meaghan Bell, Nathalie Jetté

**Affiliations:** 1 Department of Community Health Sciences and the O’Brien Institute for Public Health, Cumming School of Medicine, University of Calgary, Calgary, Alberta, Canada; 2 Department of Clinical Neurosciences and the Hotchkiss Brain Institute, Cumming School of Medicine, University of Calgary, Calgary, Alberta, Canada; 3 Department of Critical Care Medicine, Cumming School of Medicine, University of Calgary, Calgary, Alberta, Canada; 4 Department of Medicine, Cumming School of Medicine, University of Calgary, Calgary, Alberta, Canada; Taipei Medical University Shuang Ho Hospital, TAIWAN

## Abstract

**Objectives:**

Clinical practice guidelines have the potential to improve care, but are often not optimally implemented. Improving guideline use in clinical practice may improve care. The objective of this study was to identify the barriers and facilitators (determinants) of guidelines use among neurologists and to propose a strategy to improve guideline implementation.

**Methods:**

This was a mixed-methods study design. A quantitative, population-based, cross-sectional survey of Canadian neurologists was conducted. Associations between guidelines use and determinants of guidelines use were examined. Focus groups and interviews were conducted using purposeful sampling of the population. Determinants of guideline use were mapped to interventions to establish a strategy for guideline implementation among neurologists.

**Results:**

38.7% (n = 311) of neurologists responded to the survey. Typically, respondents had been practicing for 16.6 years and worked in an academic institution in an urban setting. Being male and having an academic affiliation was associated with guideline use. Determinants of guideline use differed between guideline users and non-users; non-users consistently rating determinants lower than users, especially applicability. Two focus groups and one interview (n = 11) identified six main themes of determinants of guideline use: Credibility, knowledge, applicability, resources, motivation, and target audience; which was congruent with the quantitative data. The proposed knowledge translation strategy contains three pillars: guidelines development, dissemination, and interventions.

**Conclusions:**

Several determinants of guideline use not commonly discussed in the literature were identified (applicability, target audience, credibility). The proposed implementation strategy is a valuable resource for guideline developers and policy/decision-makers to improve knowledge translation of guidelines among neurologists.

## Introduction

Remaining up-to-date with evidence-based practices within and across neurological subspecialties is increasingly challenging, due to the growing and sometimes conflicting body of evidence. Clinical practice guidelines (CPG) are systematically created documents that contain recommendations to assist health professionals optimize care, based on the evidence.[1]

In many settings, CPGs can improve care by improving processes of care and clinical outcomes.[2–4] Despite evidence of these potential benefits of CPGs in other settings, there is little evidence that this is the case in neurology. For example, the benefits of using CPGs in a common neurological condition, epilepsy, have not been observed, likely due to poor implementation of CPGs in clinical practice.[5,6] It is hypothesized that some determinants of CPG use for the care of people with neurological conditions are similar to those for CPG use in general.[7] Namely, individuals’ lack of awareness, familiarity, agreement, and motivation; environmental factors; and CPG-related factors.[7] However, the reason for the poor adherence to CPGs in neurology remains elusive.[8] Applying knowledge translation (KT) methodologies and strategies to CPGs presents an opportunity to improve dissemination and implementation of CPGs. KT is the exchange, synthesis, and application of knowledge among researchers and users to accelerate the capture of the benefits of research.[9]

The objective of this study was to identify barriers and facilitators (determinants) to the use of CPGs among Canadian neurologists. The determinants of CPG use among neurologists were then linked to theoretical and evidence-based behavior change constructs (i.e., the Theoretical Domain Framework [TDF]) to provide evidence-informed and theory-based suggestions for improving the use of CPGs. It is hypothesized that CPG use will be low and that novel determinants of CPG use will be identified.

## Materials and methods

### Study design

A mixed methods study design (quantitative survey and qualitative focus groups and interview) was used to explore the determinants of CPGs use among neurologists.

### Population-based survey (quantitative)

#### Survey development

A population-based, cross sectional survey of Canadian neurologists was conducted between April and November 2015. Demographic variables and variables related to determinants of CPG use in clinical practice were collected (S1 File). The survey was developed by the study authors based on the TDF for behavior change,[10] which has been validated and used in similar studies (i.e., to identify barriers and facilitators to the use of CPGs in Australia).[11,12] Survey responses were dichotomous, continuous (seven-point Likert scale), or open text box.

The survey was pilot tested for face validity using a convenience sample of ten neurologists. Neurologists that participated in the pilot test provided feedback on: time to complete the survey, ease of the survey, and perceived objectives of the survey through open-ended questions at the end of the survey. The mean length of time to complete the survey was seven minutes. All participants rated the survey as moderately easy or very easy to complete, and correctly identified the objective of the survey.

Since Canada is a bilingual country (English and French) survey documents were provided to neurologists in their preferred language to minimize response bias based on language. The survey was developed in English and translated to French by an experienced translator who is also a neuroscience nurse.

#### Participants

All neurologists practicing in Canada were invited to participate in the study. Neurologists were excluded if they were retired, on sabbatical/leave (maternity or other), or no longer practicing in Canada.

#### Ascertainment

Contact information (excluding email) for neurologists practicing in Canada was obtained from the Canadian Medical Directory. A modified Dillman method,[13] using a minimum of three modes of contact (mail, email, fax, phone) was employed to ascertain participants.

The survey was initially distributed by mail to all neurologists. Neurologists were offered four methods to return the survey (mail, fax, email or electronically via Adobe Form Central) and were given four weeks to complete the survey, after which a reminder was sent either via email (if publically available) or fax. Neurologists were given another four weeks to complete the survey, after which they were contacted by phone to obtain their correct contact information, and their preferred contact method for the subsequent survey distribution.

#### Data analysis

Descriptive statistics were used to describe the respondent characteristics and determinants of CPG use. Non-responders were compared to responders. All comparisons were done using Students T-tests, or *X*^2^ when the data were non-parametric.

Regression analysis was used to examine the association between demographic factors (sex, age, years of practice, academic affiliation, urban or rural practice) and the determinants of CPG use, and between CPG users and non-users. Linear regression was employed to examine relationships between determinants of CPG use that were rated on the seven-point Likert scale (Likert scales were treated as a continuous variable). Logistic regression was used to examine relationships between determinants that were evaluated using dichotomous responses. Variables that were found to be significantly different between CPG users and non-users were included in the regression model to control for effect measure modifiers and confounders.

Using data from the responders, the proportion of CPG use and determinants of CPG use were stratified based on demographic profiles (years of practice, urban or rural, general or subspecialty, academic affiliation) of “responders” and “non-responders” to help quantify the direction and magnitude of potential response biases.

All quantitative data analysis was conducted using STATA 12.[14] For all tests of significance, a p-value of <0.05 was considered statistically significant. In instances where there were multiple comparisons, an *a priori* decision was made to use a Bonferonni correction.

Qualitative data were analyzed according to the qualitative data analysis methods outlined below.

### Focus groups and interview (qualitative)

A phenomenological approach was used to understand and conceptualize how the phenomena (CPGs) are perceived by neurologists. Based on our objective to expand our understanding of factors that influence the use of CPGs and to identify any novel determinants that were not probed using the quantitative survey among neurologists (a heterogeneous group), focus groups and interview were conducted.[15–17]

#### Participants

Participants were ascertained from survey respondents and were purposefully sampled based on survey responses. Participants were selected to be representative of the population on: age, years of practice, geographical location, patient population (adult vs. pediatric, and general vs. subspecialty), and setting (academic affiliation and rural vs. urban). All participants completed a written consent form prior to the focus group or interview.

#### Data collection

A semi-structured, iterative script based on the TDF was used to guide the focus groups and interview (S2 File). The initial script was tailored to preliminary survey results. The script for the subsequent focus group and interview was tailored to the results of the initial focus group to attempt to elicit novel responses.

The same experienced facilitator moderated all focus groups and the interview. An assistant facilitator took notes and validated the transcript and analyses. A combination of a face-to-face focus group, an online/webinar focus group, and an interview were employed to maximize participation of neurologists from across the country (six time zones). Data from different methods of focus groups can be combined and is an acceptable means of data collection.[18] All focus groups and the interview were audio recorded and transcribed verbatim.

#### Data analysis

A phenomenological approach was taken to identify themes in the lived experience of neurologists using CPGs in their practice.[19,20] The facilitator transcribed all audio recordings and the assistant facilitator reviewed the transcripts for accuracy. Deductive qualitative analysis was used. The codes or “nodes” in NVivo (Mac version 10.2.2)[21] were informed by two previously published studies that examined determinants of CPG use and behaviour change (S3 File).[7,22] Data were coded into the evidence-informed nodes, which were then thematically analyzed to arrive at themes of determinants of CPG use.

The facilitator and an additional analyst with qualitative expertise independently coded the transcripts using standardized code definitions. The two analysts compared coding to ensure consistency and agreed on the themes. Quantitative data analysis was used to determine the frequency with which each theme was endorsed and the number of participants that endorsed each theme.

#### Saturation

The two analysists were identified as subject matter experts based on their expertise in the area of KT and CPGs, or qualitative methods. Saturation was defined as the absence of additional themes and was discussed after each focus group/interview. Saturation was determined through consensus among the subject matter experts.

### Knowledge translation strategy

Implementations strategies informed by theory may improve the success of the implementation;[23,24] therefore, the Knowledge to Action (KTA) framework was chosen for this study.[25] Using the KTA as our framework, the TDF[10] which was developed based on a literature review of psychological theories relevant to behavior change and consensus process among experts in the field, was employed to classify the identified determinants of CPG use to facilitate mapping the determinants to the Behaviour Change Wheel.[22] The Behaviour Change Wheel is based on the TDF and maps behaviour change interventions to determinants of CPG use in order to tailor the KT strategies.

### Standard protocol approvals, registrations, and patient consents

This study was approved by the University of Calgary Health Research Ethics Board.

## Results

### Population-based survey (quantitative)

#### Study participants

**Fig 1:**
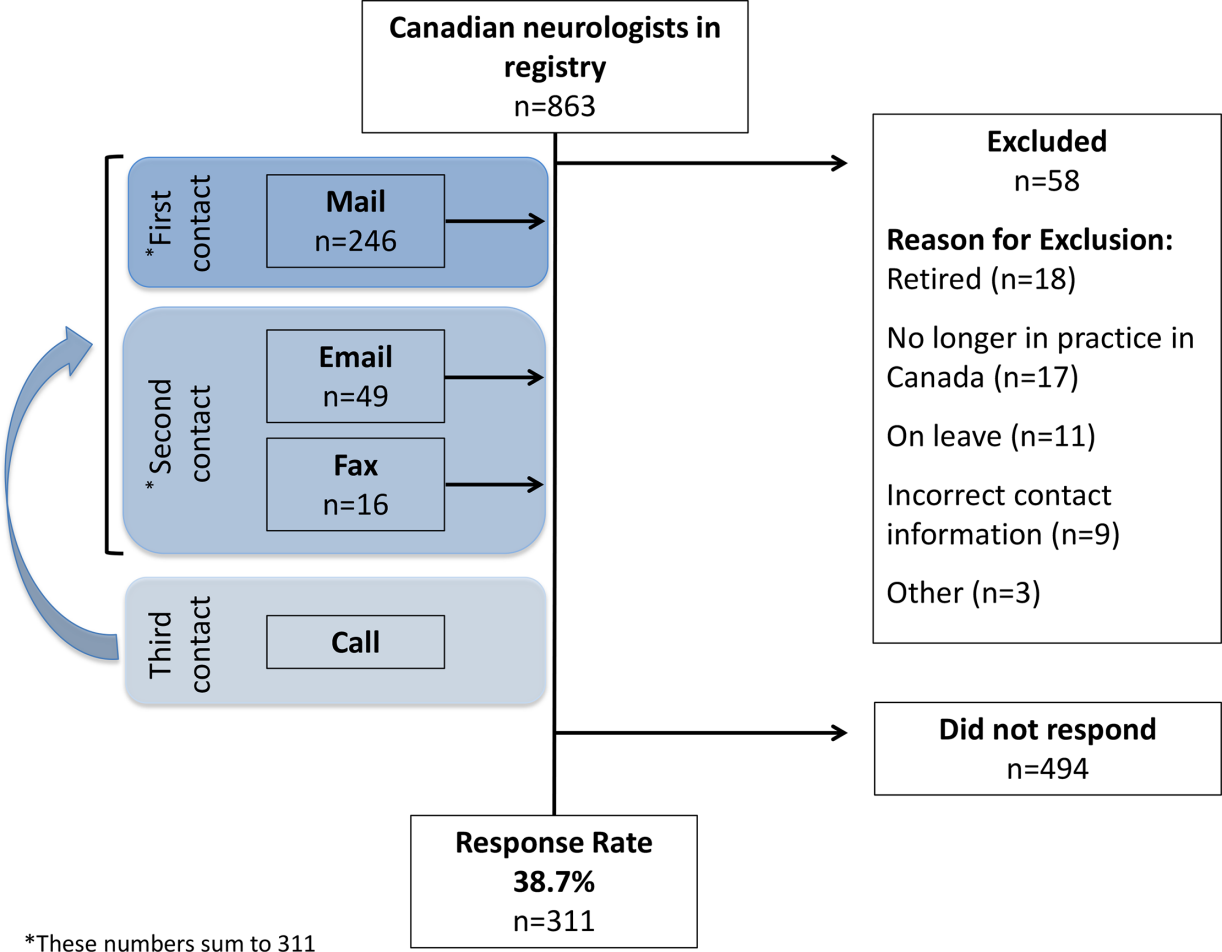
Diagram of the flow of participants through the study. Participants were first contacted by mail, then email or fax. Participants that did not respond were called and asked to provide their preferred method of contact.

The response rate for the national survey was 38.7% (n = 311; Fig 1). Typically, participants had been in practice for 16.6 years (SD = 12.2), practiced in an urban area, had an academic affiliation, and were subspecialized (Table 1). Non-responding neurologists differed from the responders on all variables except for sex and language (Table 1).

**Table 1:** Population characteristics.

	**Responders** **n = 311**	**Non-responders** **n = 501**	***X***^**2**^
**Variable**	n (%)	n (%)	
**Sex**	Male = 212 (68.2)	Male = 343 (68.5)	p = 0.76
**Urban or rural**	Urban = 287 (92.3) Rural = 12 (3.9)	Urban = 428 (85.2) Rural = 73 (14.8)	p<0.001
**Academic affiliation**	Yes = 257 (76.8)	Yes = 142 (28.1)	p<0.001
**General or subspecialty**	Subspecialty = 224 (65.7) Epilepsy n = 38 Neuromuscular n = 32 Stroke n = 32 Movement disorder n = 29 Multiple sclerosis n = 28 Headache/pain n = 17 Cognitive n = 13 Electromyography n = 10 Behavioural neurology n = 9 EEG n = 7 Neuro-oncology n = 7 Vascular n = 6 Sleep n = 5 Neuro-opthomology n = 4 Neurocritical care n = 4 Genetics n = 4 Neurometabolic n = 1 Neuroendocrinology n = 1	Subspecialty = 189 (37.3)	p<0.001
**Years of practice**	Mean = 16.6 years (SD = 12.2)	Mean = 26.2years (SD = 12.8)	t-test p<0.001
**Language**	French = 52 (16.9) English = 256 (83.1)	French = 85 (17.1) English = 413 (82.9)	P = 0.95

*Abbreviations*: **n** = sample size; **SD** = standard deviation; **X**^**2**^ = chi squared; **p** = p-value, **EEG** = electroencephalography

#### CPG use

Of the respondents, 76.5% indicated they used CPGs in their practice. The most commonly reported CPGs used by the respondents are listed in S4 File. The median response on the seven-point Likert scale to the statement “I use CPGs in my clinical practice” was 5 (IQR = 4). Sex (52.2% of males vs. 26.9% of females, p = 0.01) and academic affiliation (academic affiliation = 69.5% vs. no affiliation = 9.3%, p = 0.02) were associated with CPG use (estimated power = 1.0).

#### Determinants

Table 2 lists the determinants of CPG use examined. Overall, having the skills and resources to adhere to the recommendations in the CPGs, were not identified as barriers to the use of CPGs (4.1% and 13.8% responded yes, respectively). Conversely, lack of knowledge of CPGs was commonly (41.2%) endorsed as a barrier, as was time constraint (38.7%), and the applicability of CPGs to clinical practice (34.8%). Many of these determinants were corroborated when asked why they do not use CPGs (free-text box in the questionnaire). The barriers that were identified qualitatively included the following CPG characteristics: poor applicability (n = 24), unnecessary or unhelpful (n = 22), lack of credibility (n = 11), insufficient benefit (n = 11), unawareness or unfamiliarity with CPG (n = 7), rigidity of CPGs (n = 7), lack of accessibility (n = 5), outdated CPG (n = 5), impractical (n = 4), and time or resource constraints (n = 3).

**Table 2:** Determinants of CPG use by CPG users and non-users (univariate analysis controlling for sex and academic affiliation).

**Barrier**	All*		**CPG User**	**CPG Non-user**	
	n (% Yes)		n (% Yes)	n (% Yes)	p-value
There are incentives to follow CPGs in my practice	29 (9.7)		26 (11.0)	3 (4.8)	0.21
Lack of knowledge about CPGs is a barrier to their use in my practice	121 (41.2)		90 (39.0)	30 (48.4)	0.18
Time constraints are a barrier to the use of CPGs in my practice	115 (38.7)		85 (36.3)	29 (46.8)	0.28
The applicability of CPGs to my clinical setting is a barrier to their use	101 (34.8)		66 (28.6)	35 (60.3)	<0.001
I do not have the skills to perform the standards of care recommended in most CPGs	12 (4.1)		3 (5.0)	9 (3.9)	0.89
I do not have the resources to perform the standards of care recommended in most CPGs	40 (13.8)		30 (13.1)	10 (16.9)	0.63
**Barrier/Facilitator**					
	**All**		**CPG User**	**CPG Non-user**	
	**Median (IQR)**		**Median (IQR)**	**Median (IQR)**	**p-value**
My colleagues use CPGs in their clinical practice	5 (2)		5 (2)	3 (1)	<0.001
CPG recommendations influence my clinical practice	6 (3)		6 (2)	3 (2)	<0.001
The use of CPGs is supported in my institution	5 (2)		6 (2)	4 (2)	<0.001
It is easy to perform standards of care outlined in CPGs	5 (2)		5 (2)	4 (2)	<0.001
Recommendations are often in line with my professional opinion	6 (1)		6 (1)	5 (2)	0.001
The benefit of using CPGs outweighs the costs	4 (3)		4 (2)	3 (3)	0.12
Following CPGs improves the quality of care I deliver	5 (2)		6 (1)	4 (2)	<0.001
It is easy to remember the care plan outlined in CPGs when I see patients	5 (2)		5 (2)	3 (2)	<0.001
Using CPGs in my practice is worth the effort	5 (2)		6 (1)	3 (2)	<0.001

*Abbreviations*: **CPG** = clinical practice guideline; **IQR** = interquartile range (q75-q25); **n** = number of participants; **p-value** = p-value of comparisons between CPG users and non-users; **ns** = non-significant

*Footnote: CPG Users and CPG Non-users may not sum to equal the All group due to missing values on the CPG use question

Survey items probing determinants of CPG use in clinical practice (seven-point Likert scale ranging from barrier = 0 to facilitator = 7) were rated highly (median = 5), indicating that these variables were facilitators rather than barriers to CPG use (Table 2). The determinant rated as the greatest facilitator asked about the influence of CPGs on clinical practice (median = 6, IQR = 3). The item that had the lowest score asked about the benefits and cost of using CPGs (median = 4, IQR = 3), indicating that the benefit did not outweigh the cost of using CPGs (S1 Fig.).

Regression analysis (controlling for sex and academic affiliation since they were associated with CPG use) revealed that applicability of CPGs was the only determinant that differed between CPG non-users and CPG users (60.3% vs. 28.6%, p<0.001, S1 Table). However, CPG non-users consistently rated all determinants of CPG use lower than CPG users (Table 2).

Stratification of participants based on demographic characteristics of “responders” and “non-responders found a trend towards less CPG use among “non-responders”, but there were no differences in determinants of CPG use between the two groups (S2 Table).

### Focus groups and interview (qualitative)

#### Study participants

The first focus group was conducted in a face-to-face setting (n = 8), the second focus group was conducted using webinar software (n = 2), and the interview was conducted via telephone. The majority of focus group participants practiced in an urban setting (90.9%), had an academic affiliation (72.7%), were subspecialists (63.6%), and saw a predominantly adult population (63.6%). The mean number of years the participants were in practice was 25 year (SD = 13.4). Eighty-one percent (81%) of participants reported using CPGs in their clinical practice.

#### Determinants

From the initial 33 nodes deduced from the literature (S3 File) our data identified six themes related to determinants of CPG use: credibility, knowledge, motivation, applicability, resources, and target audience (Table 3).

**Table 3:** Focus group results.

**Theme**	**No. Participants** **(n = 11)**	**No. Ref**	**Example**
**Credibility** [agreement, bias synthesis, interpretation of evidence, confidence in CPG developer, timeliness, development, guideline factors]	10	106	“All kinds of people get together and write guidelines and you don’t have any sense of how, well, critical was the literature review and how critical and firmly the evidence base.”
**Knowledge** [education, access, awareness, familiarity, volume of information, skills, self-efficacy]	10	90	“Point of care access. Most of us generally don’t review guidelines exhaustively–it’s when we’re prompted for a challenging case. There is a time sensitive nature.”
**Motivation** [coercion, enablement, reimbursement, incentivisation, outcome expectancy, risk & benefit, modelling]	10	83	“You need to follow them or you fall into the medical legal trap of not doing what is indicated”
**Applicability** [patient & setting, challenge autonomy, rigid, guideline factors]	10	81	“I don’t think a lot of the patients I see in my daily work have an applicable guideline.”
**Resources** [environmental factors, organizational constraints, time]	11	46	“Yet the access to the infrastructure and resources necessary are diminishing…guidelines are built without recognizing that.”
**Target Audience**	7	23	“I think guidelines are good for people like me who do everything. We have a general practice and we are not specialists in epilepsy.”

*Abbreviations*: **No.** = number

Three common discussion points related to credibility included the rigor of the development process, the reputation of the organization developing and endorsing the CPG, and the CPG being up-to-date.

Many of the references to knowledge were related to awareness of and access to CPGs, especially in a timely manner (i.e., at point of care and when they are still up-to-date). Access to CPGs was also included within the resources theme, such that in some settings the resources to access CPGs were not available, especially at the point of care. Other issues related to resources included the time required to provide the recommended care outlined in CPGs.

The discussion around applicability was commonly focused on the applicability to the complex, sub-population of patients commonly seen; and also to the practice setting. Discussion around applicability was linked to the discussion around the target audience, in that CPGs were better targeted to care settings where the CPG are appropriate to the patient population. The motivation for using or seeking a CPG was often in response to a patient that fell outside of the general area of the neurologist’s practice, and in cases where it was felt that not following a CPG’s recommendations could result in penalties.

#### Saturation

Saturation of themes was reached after the second focus group; however, an additional interview was conducted to confirm saturation.

### Knowledge translation strategy

**Fig 2:**
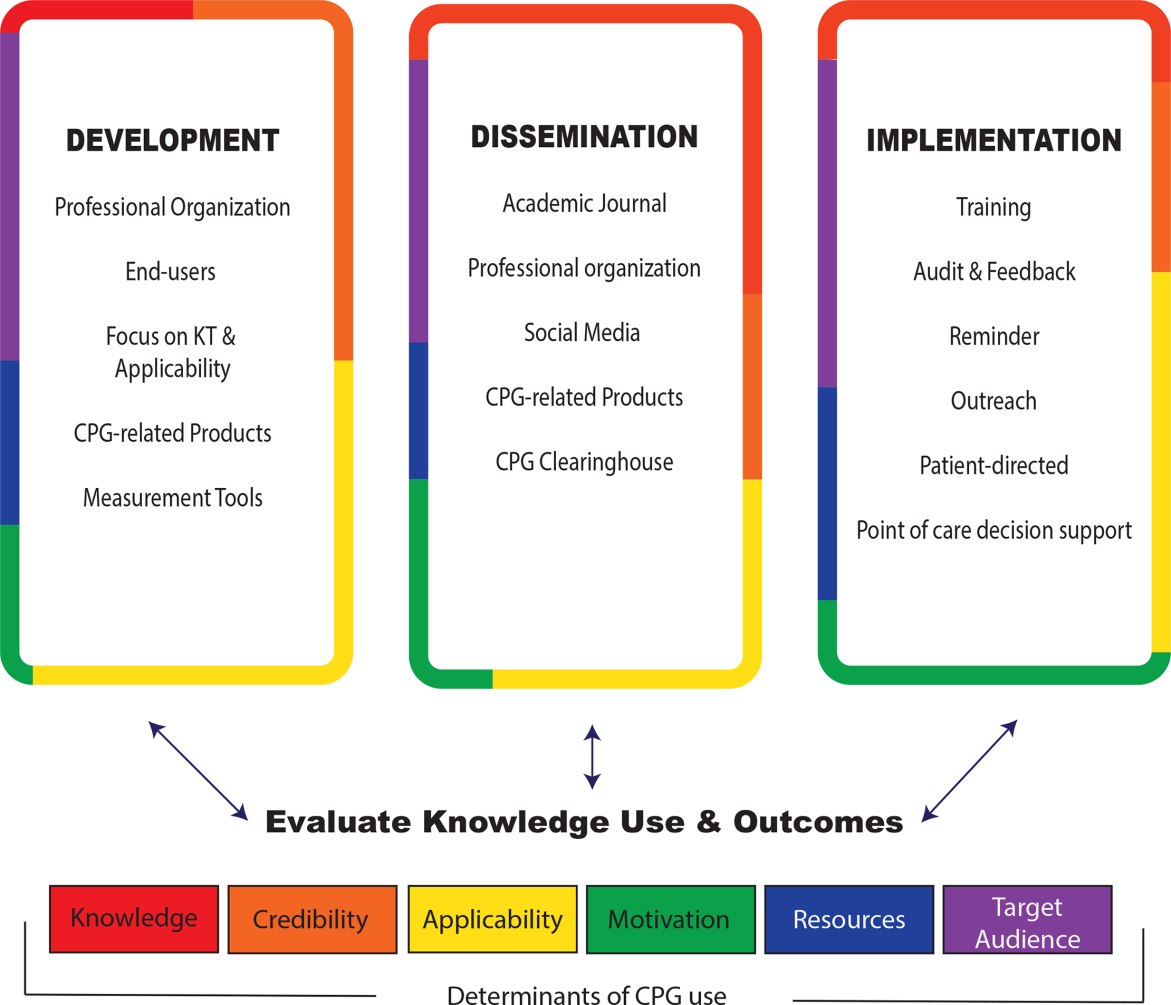
The KT strategy for guidelines. Each of the 3 pillars of the KT strategy are outlined in colours that each pillar addresses, that correspond to the determinants of CPG use that have been identified. The need to evaluate knowledge use and clinical outcomes is highlighted by the bidirectional nature of the arrows.

The evidence-based determinants of CPG use among neurologists (credibility, knowledge, motivation, applicability, resources, target audience) were linked to the Behavioural Change Wheel using the TDF (S2 Fig and S5 File) to guide the proposed KT strategy (Fig 2).[10,22] The proposed KT strategy was structured according to the KTA framework[25] and has three main pillars: 1) CPG development, 2) implementation and 3) interventions; each of which targets many determinants of CPG use identified in our study (Fig 2 and S5 File).

A detailed description of the role of each component of the KTA in the proposed KT strategy is provided in S6 File.

## Discussion

This study provides a national evaluation of determinants of CPG among neurologists and provides a multi-dimensional, evidence-informed KT strategy to improve the use of CPGs in clinical practice. Insight into the unique barriers and facilitators of CPG use in day-to-day practice among neurologists were identified (credibility, applicability, target audience), along with the strength of their association to CPG use.

Of the six themes identified here (credibility, knowledge, motivation, applicability, resources, and target audience) some were previously reported in other clinical settings, such as knowledge, which is the most commonly cited determinant.[7,22] Importantly, our study identified novel determinants that may be specific to CPG use among neurologists (credibility, applicability, and target audience). Also, our study provides quantification of which determinants are most strongly associated with implementation of CPG recommendations among neurologists (strength of association).

Applicability (to the patient population, clinical setting, and end-user) has not been frequently reported as a barrier to CPG use,[7] but was the barrier that was most strongly associated with CPG use in the quantitative component of our study. Applicability may be unique to neurologists because a high proportion of neurologists subspecialize and thus encounter complex patient populations that require individualized treatment where there is limited evidence to guide care, rendering CPG recommendations inapplicable. Applicability as a barrier to CPG use is echoed in a recent systematic review of CPGs for epilepsy in which the applicability of existing CPGs was poor according to the AGREE II tool.[26] Improving CPG development methodology may present an opportunity to improve the applicability of CPGs.

Credibility was associated with CPG use in both the qualitative and quantitative data. Factors related to credibility as a barrier included biased synthesis and interpretation of the evidence, and lack of rigorous methodologies. Conversely, if the CPG was developed and disseminated by a reputable professional organization, it facilitated CPG use. For example, among our sample of neurologists, CPGs developed by the American Academy of Neurology were noted as credible and were cited as the CPGs more commonly used in clinical practice.

The present study used a mixed-methods design, which was a strength and provided rich understanding of the determinants of CPG use and the ability to examine congruency between the quantitative and qualitative data, as a means of validating each data set.[27] Congruency between the quantitative and qualitative data was observed between many of the variables and themes in this study.

While the design is a study strength, there are also limitations to consider when interpreting our findings. First, we achieved a response rate of nearly 40%, which is arguably low, but higher than reported in other studies.[28] Our ascertainment methods also yielded enough cases to achieve adequate power to examine the strength of the associations between CPG use and determinants of their utilization–a unique aspect of this study. Secondly, demographic differences between responders and non-responders were identified, indicating a potential bias. These differences could partially be explained by the fact that the demographic data for non-responders were taken from a database, while data for the responders were self-reported. Based on the analysis comparing demographic profiles of non-responders to demographic profiles of responders, we identified that our findings present a moderately optimistic picture of CPG use among neurologists–CPG use would likely be slightly lower among those who did not respond but determinants would be similar. This potential bias could limit the generalizability of our data beyond academic, subspecialty neurologists practicing in an urban area with more than 17 years of practice. A recent national survey of neurologists demonstrated that only a small portion of neurologists are in private, non-academic practices[29] suggesting our sample represents the majority of Canadian neurologists. However, this minority were more likely to not respond and are an important target audience for CPG use, which highlights a gap in our knowledge of CPG use and determinants of CPG use among general neurologists with many years of experience in community practice in rural areas. Future research should aim to find methods to engage this population to identify CPG use and determinants of CPG use among this group.

## Conclusion

While CPGs are not always viewed favorably among physicians,[30] they have the capacity to improve quality of care when effectively implemented.[2,4] For example, mortality was reduced and treatment efficacy was increased among those who were compliant with stroke CPGs.[31] However, CPGs are often not implemented or adopted in day-to-day practice in neurology.[5,6,32] Developing a CPG is a resource intensive undertaking that can be wasteful if recommendations within CPGs are not adopted into clinical practice. This study provides a multi-faceted, tailored KT strategy for implementing CPGs among neurologists; these factors have been found to increase the effectiveness of KT strategies.[23–25,33] Improving implementation of recommendations within high quality CPGs through KT interventions may decrease variation in practice, consequently improving the quality of care provided to patients with neurological conditions.

## Supporting information

S1 File Survey of neurologists regarding barriers and facilitators to using clinical practice guidelines (English and French). (PDF)

S2 File Focus group guide. (PDF)

S3 File Code book for qualitative analysis of focus groups. (PDF)

S4 File Clinical practice guidelines used by neurologists. (PDF)

S5 File Expanded mapping of determinants of guideline use among neurologists to Theoretical Domains Framework and intervention functions to the proposed knowledge translation strategy. (PDF)

S6 File Detailed knowledge translation strategy. (PDF)

S1 Fig Determinants of clinical practice guideline use by neurologist. (PDF)

S2 Fig Mapping determinants of guideline use among neurologists to Theoretic Domains Framework and intervention functions. (JPG)

S1 Table Factors associated with guideline use among neurologists. (PDF)

S2 Table CPG use and determinants of CPG use for profiles of responders and non-responders. (PDF)
