# Supplementary material for: Knowledge translation of clinical practice guidelines among neurologists: A mixed-methods study
Source: PLoS One. 2018 Oct 10;13(10):e0205280. doi: 10.1371/journal.pone.0205280 (PMC6179253; doi:10.1371/journal.pone.0205280)
Supplement: S1 File — (PDF) [file pone.0205280.s001.pdf]

Study ID:

Clinical Practice Guideline Questionnaire for Neurologists

1. In what country do you practice Neurology?

- ☐ Canada
- ☐ Other, please specify:

2. Do you practice Neurology in a rural or urban setting?

- ☐ Urban
- ☐ Rural

3. Are you affiliated with a tertiary care academic institution (ie: university)?

- ○ Yes
- ○ No

4. What is your area of clinical practice? (check all that apply)

- ☐ General neurology
- ☐ Subspecialty neurology, please specify below
- ☐ Other, please specify:

Please specify subspecialty

5. For how many years have you been a practicing neurologist?

6. In what country did you complete your neurology residency training?

7. Which patients do you see in your practice? Check all that apply.

- ☐ Adult
- ☐ Pediatric

8. Do you use clinical practice guidelines in your practice?

- ○ Yes
- ○ No

I***f yes***, which guidelines do you routinely refer to (up to 5)?

If no, why do you not use clinical practice guidelines?

9. Please indicate how much you agree or disagree with the following statements.

	1 Strongly Disagree	2	3	4	5	6	7 Strongly Agree	N/A
I use clinical practice guidelines in my clinical practice.	○	○	○	○	○	○	○	○
My colleagues use clinical practice guidelines in their clinical practice.	○	○	○	○	○	○	○	○
Clinical practice guideline recommendations influence my clinical practice.	○	○	○	○	○	○	○	○
The use of clinical practice guidelines is supported in my institution.	○	○	○	○	○	○	○	○
It is easy to perform the standard of care recommended in clinical practice guidelines.	○	○	○	○	○	○	○	○

	1 Strongly Disagree	2	3	4	5	6	7 Strongly Agree	N/A
Recommendations are often in line with my professional opinion.	◯	◯	◯	◯	◯	◯	◯	◯
In general, the cost of following the recommendations in clinical practice guidelines outweigh the benefits.	◯	◯	◯	◯	◯	◯	◯	◯
Following clinical practice guidelines improves the quality of care I deliver.	◯	◯	◯	◯	◯	◯	◯	◯
It is easy to remember the care plan outlined in clinical practice guidelines when I see patients.	◯	◯	◯	◯	◯	◯	◯	◯
Using clinical practice guidelines in my practice is worth the effort.	◯	◯	◯	◯	◯	◯	◯	◯

The following questions ask about factors that may be barriers to implementing clinical practice guidelines in your practice.

10. Are there incentives to following clinical practice guidelines where you practice?

◯ Yes

◯ No

If yes, what are the incentives?

11. Lack of knowledge about clinical practice guidelines is a barrier to their use in my practice.

◯ Yes

◯ No

12. Time constraints are a barrier to the use of clinical practice guidelines in my practice.

◯ Yes

◯ No

13. The applicability of clinical practice guidelines to my clinical setting is a barrier to their use.

- ◯ Yes
- ◯ No

14. I do not have the skills to perform the standards of care recommended in most clinical practice guidelines.

- ◯ Yes
- ◯ No

15. I do not have the resources to perform the standards of care recommended in most clinical practice guidelines.

- ◯ Yes
- ◯ No

16. Are there any other barriers to implementing clinical practice guidelines in your practice?

17. What are some facilitators to implementing clinical practice guidelines in your practice?

18. Do you see any patients with seizures/epilepsy?

- ◯ No - if no, thank you for your participation. This survey is now complete.
- ◯ Yes - if yes, please answer the questions below

19. Approximately how many patients with epilepsy do you treat each month?

- ◯ < 5
- ◯ 5 - 19
- ◯ 20 - 40
- ◯ > 40

20. Is there an epilepsy program in your city?

- ◯ Yes
- ◯ No
- ◯ Don't know

21. Please indicate how much you agree or disagree with the following statements.

	1 Strongly Disagree	2	3	4	5	6	7 Strongly Agree	N/A
I feel confident treating persons with epilepsy.	○	○	○	○	○	○	○	○

22. Are you aware of any clinical practice guidelines for ***the care of people with epilepsy***?

- ○ Yes
- ○ No

a) ***If yes***, how many clinical practice guidelines ***for the care of people with epilepsy*** are you familiar with?

- ○ 1 - 5
- ○ 6 - 10
- ○ 11 - 20
- ○ > 20

b) ***If yes***, which clinical practice guidelines ***for the care of people with epilepsy*** do you use in your practice?

23. Please use the space below to add any additional thoughts or comments regarding the use of clinical practice guidelines for clinical care. Feel free to use the back of this page also if necessary.

24. Are you willing to participate in a focus group examining the barriers and facilitators of epilepsy guidelines?

- ○ Yes
- ○ No

If yes, would you be able to attend the focus group online or by telephone?

- ○ Yes
- ○ No

We sincerely thank you for your participation.

Indentification D'Étude:

☐

Questionnaire sur les guides de pratique à l’intention des neurologues

1. Dans quel pays practiquez-vous la neurologie?

☐ Canada
☐ Autre, spécifier: ☐

2. Pratiquez-vous la neurologie dans en milieu urbain ou rural?

☐ Urbain
☐ Rural

3. Êtes-vous rattaché/rattachée à un établissement d'enseignement de soins tertiaires (c'est-à-dire à une université)?

○ Oui
○ Non

4. Quel est votre champ de pratique clinique (cochez toutes les cases appropriées)?
Neurologie générale

☐ Neurologie générale
☐ Neurologie - surspécialité (précisez laquelle plus bas)
☐ Autres (précisez): ☐

Surspécialité (précisez laquelle ici):

☐

5. Depuis combien d’années pratiquez-vous la neurologie?

☐

6. Dans quel pays avait-vous fait votre résidence en neurologie?

☐

7. Quel type de patients voyez-vous dans votre pratique (cochez toutes les cases appropriées)?

☐ Adultes
☐ Enfants

8. Utilisez-vous des guides de pratique clinique dans votre pratique?

○ Oui
○ Non

Si oui, à quels guides vous reportez-vous habituellement (nommez-en jusqu'à 5)?

Si non, pourquoi n'utilisez-vous pas des guides de pratique clinique?

9. Veuillez indiquer dans quelle mesure vous êtes d’accord ou non sur les énoncés suivants.

	1 Pas du tout d'accord	2	3	4	5	6	7 Tout à fait d’accord	S.O.
J’utilise des guides de pratique clinique dans ma pratique.	◯	◯	◯	◯	◯	◯	◯	◯
Mes collègues utilisent des guides de pratique clinique dans leur pratique.	◯	◯	◯	◯	◯	◯	◯	◯
Les recommandations contenues dans les guides de pratique clinique modifient ma pratique.	◯	◯	◯	◯	◯	◯	◯	◯
On favorise l’utilisation des guides de pratique clinique dans mon établissement.	◯	◯	◯	◯	◯	◯	◯	◯
Les normes de soins recommandées dans les guides de pratique clinique sont faciles à appliquer.	◯	◯	◯	◯	◯	◯	◯	◯
Les recommandations sont souvent dans le même sens que mon avis de professionnel.	◯	◯	◯	◯	◯	◯	◯	◯

	1<br>Pas du tout d'accord	2	3	4	5	6	7<br>Tout à fait d'accord	S.O.
En général, les coûts liés à l'application des recommandations contenues dans les guides de pratique clinique l'emportent sur les avantages.	◯	◯	◯	◯	◯	◯	◯	◯
L'application des guides de pratique clinique améliore la qualité des soins que je donne.	◯	◯	◯	◯	◯	◯	◯	◯
Les plans de soins décrits brièvement dans les guides de pratique clinique sont faciles à se rappeler quand je vois des patients.	◯	◯	◯	◯	◯	◯	◯	◯
L'utilisation des guides de pratique clinique dans ma pratique vaut l'effort.	◯	◯	◯	◯	◯	◯	◯	◯

Les questions suivantes portent sur des facteurs susceptibles d'être des obstacles à l'application des guides de pratique clinique dans votre pratique.

10. Y a-t-il des mesures incitatives à l'application des guides de pratique clinique dans votre lieu de pratique?

◯ Oui

◯ Non

Si oui, quelles sont ces measures incitatives?

11. Le manque de connaissances sur les guides de pratique clinique constitue un obstacle à leur utilisation dans ma pratique.

◯ Oui

◯ Non

12. Les contraintes de temps constituent un obstacle à l'utilisation des guides de pratique clinique dans ma pratique.

◯ Oui

◯ Non

13. Le manque d'applicabilité des guides de pratique clinique à mon milieu clinique constitue un obstacle à leur utilisation.

◯ Oui

◯ Non

14. Je n'ai pas la compétence nécessaire pour appliquer les normes de soins recommandées dans la plupart des guides de pratique clinique.

◯ Oui

◯ Non

15. Je n'ai pas les ressources nécessaires pour appliquer les normes de soins recommandées dans la plupart des guides de pratique clinique.

◯ Oui

◯ Non

16. Y a-t-il d'autres obstacles à l'application des guides de pratique clinique dans votre pratique?

17. Quels facteurs facilitent l'application des guides de pratique clinique dans votre pratique?

18. Voyez-vous des patients qui sont atteints d’épilepsie ou qui font des crises épileptiques?

- ◯ Oui – Veuillez répondre aux questions suivantes.
- ◯ Non – Merci de votre participation. L’enquête prend fin ici.

19. Combien de patients atteints d’épilepsie traitez-vous environ par mois?

- ◯ < 5
- ◯ 5 - 19
- ◯ 20 - 40
- ◯ > 40

20. Existe-t-il un programme de prise en charge de l’épilepsie dans votre ville?

- ◯ Oui
- ◯ Non
- ◯ Je ne le sais pas

21. Veuillez indiquer dans quelle mesure vous êtes d’accord ou non sur l’énoncé suivant.

	1 Pas du tout d'accord	2	3	4	5	6	7 Tout à fait d’accord
Je me sens confiant dans la traitment des personnes atteintes d'épilepsie	◯	◯	◯	◯	◯	◯	◯

22. Connaissez-vous des guides de pratique clinique sur les soins à donner aux personnes atteintes d’épilepsie?

- ◯ Oui
- ◯ Non

a) Si oui, combien de guides de pratique clinique sur les soins à donner aux personnes atteintes d’épilepsie connaissez-vous bien?

- ◯ 1 - 5
- ◯ 6 - 10
- ◯ 11 - 20
- ◯ > 20

b) Si oui, quels guides de pratique clinique sur les soins à donner aux personnes atteintes d’épilepsie utilisez-vous dans votre pratique?

23. Veuillez utiliser l’encadré ci-dessous pour nous faire part de réflexions ou d’observations sur l’utilisation des guides de pratique clinique dans la prestation des soins cliniques. Au besoin, utilisez le verso de la page.

24. Êtes-vous disposé/disposée à participer à un groupe de discussion sur les obstacles ou les facteurs favorables à l’application des lignes directrices sur la prise en charge de l’épilepsie?

- ◯ Oui
- ◯ Non
