# Supplementary material for: Knowledge translation of clinical practice guidelines among neurologists: A mixed-methods study
Source: PLoS One. 2018 Oct 10;13(10):e0205280. doi: 10.1371/journal.pone.0205280 (PMC6179253; doi:10.1371/journal.pone.0205280)
Supplement: S2 File — (PDF) [file pone.0205280.s002.pdf]

Epilepsy Guidelines Focus Group #1

Introduction:

Welcome everyone, thank-you for taking the time to join our discussion around epilepsy guidelines. My name is ____; I am a PhD candidate and am coordinating this study. I will be the facilitator of this discussion. Our other team member is our research assistant, ____. The primary investigators are Dr. Samuel Wiebe and Dr. Nathalie Jetté.

You have been invited here today because we are interested in hearing your feedback on clinical practice guidelines from your experience with using them in your clinical practice.

We are here today to discuss clinical practice guidelines for the care of people with epilepsy. Clinical practice guidelines contain evidence-based recommendations to facilitate clinical care. While clinical practice guidelines exist for many different clinical populations and settings today we are interested specifically for clinical practice guidelines for people with epilepsy.
The evidence is that clinical practice guidelines can improve the process of care and clinical outcomes of patients. There is equally as much evidence that while clinical practice guidelines can be beneficial for patient care they are rarely adopted by physicians and even less often actually change clinical practice.
Our research group, which is made up of clinicians and researchers, is interested in understanding why clinical practice guidelines are not implemented in clinical practice and what, if any, changes would facilitate the use of clinical practice guidelines in clinical practice.

Before we begin, I would like to make sure that everyone knows that there are no right or wrong answers in our discussion today. Please share your point of view whether it differs from what other participants have shared or adds similar information. We are interested in all comments, so we are taking detailed notes. We are also audio-recording today's session because we don't want to miss any of your comments and feedback. Please be assured that all of your comments will be kept confidential and any information you provide will be kept in a locked cabinet or stored in a password protected folder on a secure server, accessible only by members of the research team. We would also like to ask everyone to respect the privacy and maintain the confidentiality of the other group members by not repeating anything that is said in today's session.

Please keep in mind that we are interested in both your positive and negative comments about clinical practice guidelines. And most importantly, we would like to hear from each of you.

Feel free to get up and get refreshments if you would like.

The session will end at 6PM.

Let’s begin by going around the table to introduce ourselves. Please let everyone know your first name and your subspecialty. Thank you.

Ask the Group:

1. From the survey responses, it seems that many of you use clinical practice guidelines in your practice. How easy is it to use clinical practice guidelines in your clinical practice?
2. What problems have you encountered when trying to follow the recommendations for clinical care in guidelines?
   a. Are the necessary resources available to you to use guidelines in your clinical practice?
   b. Is there anything about your work environment (physical environment, peers, and managers) that support or encourage the use of clinical practice guidelines?
3. Are the recommendations made in the guidelines in line with your current clinical practice?
4. What are some ways that you can envision of encouraging the use of clinical practice guidelines?
5. What guidelines are you aware of for the care of people with epilepsy and what do you think about them?
6. Is using guidelines when caring for people with epilepsy a common practice at your institution?
7. What about clinical practice guidelines for epilepsy, specifically, would facilitate the use of epilepsy guidelines in your practice?
8. What is the best way to disseminate guidelines to neurologists?

Summary and Wrap Up:

So now that we have talked about …., I would just like to take a moment and summarize what we’ve talked about today. <Summarize key points>.

Ask the Group:

Is there anything else that anyone would like to add that we may not have captured in our discussions or in our summaries?

Thank them for coming.

Epilepsy Guidelines Focus Group #2 and Interview

Introduction:

Welcome everyone, thank-you for taking the time to join our discussion around epilepsy guidelines. My name is _____, I am a PhD candidate and am coordinating this study. I will be

the facilitator of this discussion. Our other team members include _______, _______ and _________ and the primary investigators are Dr. Samuel Wiebe and Dr. Nathalie Jetté.

You have been invited here today because we are interested in hearing your feedback on clinical practice guidelines from your experience with using them in your clinical practice.

We are going to start today with an overview of the research group, and this research project.

We are here today to discuss clinical practice guidelines for the care of people with epilepsy. Clinical practice guidelines contain evidence-based recommendations to facilitate clinical care. While clinical practice guidelines exist for many different clinical populations and settings today we are interested specifically for clinical practice guidelines for people with epilepsy.

Evidence shows that clinical practice guidelines can improve the process of care and clinical outcomes of patients, but there is equally as much evidence that they are rarely adopted by physicians and even less often actually change clinical practice.

Our research group, which is made up of clinicians and researchers, is interested in understanding why clinical practice guidelines are not implemented in clinical practice and what, if any, changes would facilitate the use of clinical practice guidelines in clinical practice.

This is the second focus group that we have conducted for this study. During the first focus group the issues that were most commonly discussed were access to the guidelines, credibility of guidelines, and applicability of the guidelines to clinical populations. Today we'd like to focus our attention on other issues related to guidelines. I will ask a few questions related to these issues to help guide our discussion.

Before we begin, I would like to make sure that everyone knows that there are no right or wrong answers in our discussion today. Please share your point of view whether it differs from what other participants have shared or adds similar information. We are interested in all comments, so we are taking detailed notes. We are also audio-recording today's session because we don't want to miss any of your comments and feedback. Please be assured that all of your comments will be kept confidential and any information you provide will be kept in a locked cabinet or stored in a password protected folder on a secure server, accessible only by members of the research team. We would also like to ask everyone to respect the privacy and maintain the confidentiality of the other group members by not repeating anything that is said in today's session.

Please keep in mind that we are interested in both your positive and negative comments about clinical practice guidelines. And most importantly, we would like to hear from each of you.

Does anyone have any questions before we begin? The session will end at ________.

Let's begin by going around the table to introduce ourselves. Please let everyone know your first name. Thank you.

Ask the Group:

9. How easy is it to use clinical practice guidelines in your clinical practice?
10. What would prompt you to use a guideline in your clinical practice?
11. What problems have you encountered when trying to follow the recommendations for clinical care in guidelines?
12. Are the necessary resources available to you to use guidelines in your clinical practice?
13. What are some ways to encouraging the use of clinical practice guidelines among neurologists?
14. What guidelines are you aware of for the care of people with epilepsy and what do you think about them?
15. Do you use clinical practice guidelines when caring for people with epilepsy?
16. What is the best way to disseminate epilepsy guidelines to neurologists?

Summary and Wrap Up:

So now that we have talked about …., I would just like to take a moment and summarize what we've talked about today. <Summarize key points>.

Ask the Group:

Is there anything else that anyone would like to add that we may not have captured in our discussions or in our summaries?

Thank them for coming.
