# Supplementary material for: Knowledge translation of clinical practice guidelines among neurologists: A mixed-methods study
Source: PLoS One. 2018 Oct 10;13(10):e0205280. doi: 10.1371/journal.pone.0205280 (PMC6179253; doi:10.1371/journal.pone.0205280)
Supplement: S3 File — (PDF) [file pone.0205280.s003.pdf]

1. **Agreement:** Agreement with the guidelines and recommendations made within the guidelines.
    a. **Applicability to patient:** Do guidelines apply to patients seen in clinical practice?
    b. **Bias synthesis:** Is the synthesis of evidence and formulation of recommendations done with bias?
    c. **Challenge to autonomy:** Do the guidelines and recommendations challenge autonomy of clinical practice?
    d. **Interpretation of evidence:** Was the evidence used to develop the guideline interpreted properly?
    e. **Confidence in guideline developer:** Is there confidence in the group or organization developing the guidelines?
    f. **Not practical:** The guidelines are not practical for various reasons – resources, knowledge, accessibility, don't apply to patients.
    g. **Rigidity:** Guidelines are too rigid or not rigid enough to apply in clinical practice.
2. **Coercion:** Physicians feel coerced into using guidelines. This can be either a facilitator or barrier to use.
3. **Credibility:** Credibility of the guideline can refer to the credibility of the group developing the guideline or the evidence used to develop the guideline, or both.
4. **Education:** The guideline as an educational tool or education about guidelines, a barrier or facilitator to their use
5. **Enablement:** There are factors that enable the implementation of q guideline. These can be either guideline factors (such as prompts to the recommendations made in the guideline or reimbursement based on delivering care outlined in the guideline) or enablement of the implementation of the care outlined in the guideline (such as availability of resources to provide the care outlined in the guideline). Also defined as creating means and reducing barriers to the use of guidelines.
6. **Environmental factors:** The factors in the physicians' environment that either facilitate or impede the use of guideline in their clinical practice.
    a. **Reimbursement:** Physicians are reimbursed for delivering care outlined in the guidelines. Or, the opposite – they will not be reimbursed for services provided that are not recommended in the guideline.
    b. **Resource availability:** This may refer to the availability of resources to be aware of, and identify the required guidelines (such as access to online guideline clearing houses), or the resources required to implement the care outlined in the guideline (such as the available diagnostic tests or medications).
    c. **Time:** This refers to the time required to become familiar with the care standards outline in the guidelines (be aware of the existence of the guidelines, and to become familiar with the recommended care in the guidelines) or the time to enact the recommended standards of care in clinical practice.

    d. **Organizational constraints:** The barriers or facilitators at an organizational level. For example, the culture of adhering to best practice or evidence-based medicine at the institution, and the availability of resources (as described in "resource availability").

7. **Guideline factors:** This is a broad theme that describes factors related to the guidelines themselves that may impact their applicability to clinical practice. For example, the language used may not be clear, the methods for developing the guideline may not be rigorous, the dissemination of the guideline may not be adequate.
8. **Incentivization:** This refers to the increasing use of guidelines through either financial or other incentives to create an expectation of reward. For example, reimbursing physicians only when they show evidence of adhering to the recommended care outlined in the guideline.
9. **Knowledge:** Knowledge of the guidelines and the content of the guidelines.
    a. **Accessibility:** Does the ease of accessing the guidelines facilitate or impede the use of guidelines in clinical practice? Access refers to the ability to get the guidelines that the physician is aware of.
    b. **Awareness:** Does the awareness of the guidelines facilitate or impede the use of guidelines in clinical practice? Awareness refers to the physicians being aware of the presence of existing guidelines for a clinical area.
    c. **Familiarity:** Does the familiarity with the content of the guidelines and recommended care facilitate or impede the use of guidelines in clinical practice? Familiarity refers to how familiar physicians are with the content of the guideline, not just its existence.
    d. **Volume of information:** Volume of information can refer to the excess of information or lack thereof. The volume of information may also refer to the volume (excess or lack) of availability of guidelines for a clinical area.
10. **Modeling:** Is the use of guidelines or the recommended care modeled (providing and example for people to aspire or imitate) by colleagues; and does this encourage or impede the use of guidelines in clinical practice.
11. **Motivation:** Is there motivation to use guidelines in clinical care and does this facilitate or impede their use?
12. **Outcome Expectancy:** Does the expected outcome of using guidelines either facilitates or impedes the use of guidelines in clinical practice? For example, if the physician knows that using a guideline in clinical practice will improve the patient's outcome they may be more likely to use the guideline in their clinical practice.
13. **Patient Factors:** Patient factors which can impede or facilitate the use of guidelines in clinical practice can include, patients' preference for a certain treatment modality, or the "fit" of the patient to the population outlined in the guideline.
14. **Persuasion:** Refers to the use of communication to encourage or discourage the use of guidelines in clinical practice.
15. **Risk and Benefit:** There is a risk of not using the guideline and recommended care (for example, the risk of not adhering to the guideline for status epilepticus versus the risk of not adhering to the guideline for first seizure is substantially

more due to the potentially dire outcomes). Conversely, the benefit of following the care outlined is substantially greater than the risk.

16. **Self-Efficacy: T**he belief that the physician can perform the recommended care outlined in the guideline either encourages or impedes the adherence to guidelines in clinical practice.
17. **Skills:** The physician has the skills to provide the recommended care outlined in the guideline.
18. **Target Audience:** The target audience of the guideline either facilitates or impedes the use of the guideline. Factors related to the target audience could include the language used for the target audience, the patient-related factors for the target audience, or skills related to the target audience.
