# Supplementary material for: Knowledge translation of clinical practice guidelines among neurologists: A mixed-methods study
Source: PLoS One. 2018 Oct 10;13(10):e0205280. doi: 10.1371/journal.pone.0205280 (PMC6179253; doi:10.1371/journal.pone.0205280)
Supplement: S4 File — (PDF) [file pone.0205280.s004.pdf]

| **Organization** | **N** | **CPGs** |
|---|---|---|
| American Academy of Neurology | 117 | First seizure (n=16)<br>Headache, general (n=13)<br>Concussion (n=9)<br>Women with epilepsy (n=9)<br>Parkinson's disease, general (n=7)<br>Developmental delay (n=6)<br>Infantile spasm (n=6)<br>Essential tremor (n=6)<br>Neuropathy, general (n=5)<br>Stroke, general (n=5)<br>Muscular dystrophy (n=5)<br>Amyotrophic lateral sclerosis (n=4)<br>Epilepsy, general (n=3)<br>Epilepsy surgery (n=2)<br>Brain death (n=2)<br>Guillian Barre Syndrome (n=2)<br>Cannabis use (n=1)<br>Cerebral palsy (n=1)<br>Dementia (n=1)<br>Huntington's disease (n=1)<br>Status epilepticus (n=1)<br>Did not specify (n=13) |
| Canadian Heart and Stroke Foundation | 51 | |
| American Heart Association/ American Stroke Association | 32 | |
| Canadian Headache Society | 19 | |
| Local guidelines | 16 | |
| Canadian Neurological Science Federation | 12 | |
| Canadian Parkinson's Disease | 11 | |
| International League Against Epilepsy | 8 | |
| Canadian Multiple Sclerosis Society | 7 | |
| National Institute of Health Excellence (United Kingdom) | 7 | |
| Canadian consensus conference on diagnosis and treatment of dementia | 6 | |
| American Epilepsy Society | 5 | |
| American Association of Neuromuscular & Electrodiagnostic Medicine | 5 | |
| American College of Chest Physicians | 5 | |
| Canadian Neuropathic Pain Society | 5 | |

| | | |
|---|---|---|
| Canadian Medical Association | 4 | |
| European Federation of Neurological Sciences | 4 | |
| American Association of Pediatrics | 3 | |
| American Clinical Neurophysiologist | 2 | |
| International Headache Association | 2 | |
| Scottish Intercollegiate Guideline Network | 1 | |
| Other* | 44 | |

*Other refers to those that were not relate to neurology or referred only to a condition for which the neurologists reported using CPGs.

*Footnote:* The topic of the CPGs for the organizations other than the American Academy of Neurology were not listed.
