# Supplementary material for: Knowledge translation of clinical practice guidelines among neurologists: A mixed-methods study
Source: PLoS One. 2018 Oct 10;13(10):e0205280. doi: 10.1371/journal.pone.0205280 (PMC6179253; doi:10.1371/journal.pone.0205280)
Supplement: S5 File — (PDF) [file pone.0205280.s005.pdf]

Appendix E: Expanded KT strategies based on KT framework

**Determinant**	**Theoretical Behavioral Domain**	**Intervention Function**	**KT Strategy**
**Knowledge**	Opportunity<br>Capability	Training<br>Restriction<br>Environmental Restructuring<br>Enablement	• Target dissemination to the end user (consider language, mode of delivery, & source of dissemination)<br>• Make CPGs available at point of care<br>• CPG developer driven training modules (i.e. online)<br>• Site-specific training (CME & skills development)
**Credibility**	Motivation	Persuasion<br>Incentivization<br>Coercion<br>Training<br>Environmental Restructuring<br>Modeling<br>Enablement	• Develop CPG using rigorous methodology<br>• Dissemination by credible professional organization<br>• Recruit a respected stakeholder to act as CPG "champion"<br>• Use audit and feedback
**Applicability**	Motivation	Persuasion<br>Incentivization<br>Coercion<br>Training<br>Environmental Restructuring<br>Modeling<br>Enablement	• Improve applicability of CPGs during development phase<br>○ Ensure CPG is for a condition/population in need<br>○ Include end user<br>○ Ensure recommendations are actionable<br>• Adapt CPGs to local context<br>○ Engage end users in the adaptation process
**Motivation**	Motivation	Persuasion<br>Incentivization	• Provide incentives<br>○ Monetary

		Coercion<br>Training<br>Environmental Restructuring<br>Modeling<br>Enablement	○ Audit and Feedback<br>• Track improvement in quality of care (processes of care and clinical outcomes) through the use of CPG-based performance metrics
**Resources**	Capability, Opportunity	Training<br>Restriction<br>Environmental Restructuring<br>Enablement	• Ensure point-of-care access to CPGs<br>• Ensure the resources (human and material) are available for physicians to deliver the recommended care
**Target Audience**	Motivation	Persuasion<br>Incentivization<br>Coercion<br>Training<br>Environmental Restructuring<br>Modeling<br>Enablement	• Ensure there is a respected and well regarded CPG "champion" to enable their use<br>• Provide audit and feedback to show change in quality of care with CPG use<br>• Improve applicability of CPGs during development phase<br>○ Include end user<br>○ Ensure recommendations are actionable<br>• Adapt CPGs to local context<br>• Engage end users in the adaptation process
