# Supplementary material for: Knowledge translation of clinical practice guidelines among neurologists: A mixed-methods study
Source: PLoS One. 2018 Oct 10;13(10):e0205280. doi: 10.1371/journal.pone.0205280 (PMC6179253; doi:10.1371/journal.pone.0205280)
Supplement: S6 File — (PDF) [file pone.0205280.s006.pdf]

**Appendix F:** Expanded results for KT strategy by KTA

Knowledge creation

Incorporating the knowledge creation funnel into the KT strategy, in particular to the development of CPGs, is important to facilitate the dissemination and implementation of knowledge into practice. This study demonstrates that among neurologists, credibility and applicability of CPGs are key determinants of CPG use. However, applicability of some CPGs aimed towards neurologists has been found to be moderate,[,1] suggesting applicability is a barrier of CPG use clinically among neurologists. Credibility and applicability as barriers to CPG use can be overcome through improving the methodology to develop CPGs. CPG development should use rigorous methodology, while ensuring that the clinical questions addressed in the CPG are relevant, and that KT of the CPG is considered throughout the CPG development. Additionally the CPG end user should be included in the development process, as physicians are more likely to adopt a CPG into clinical practice if they were involved in the development of the CPG.[2]

Intervention strategies aimed at the CPG development process link to the following determinants of CPG use among neurologists identified in our study: credibility, applicability, and target user.

Adaptation to the local context

To adapt the knowledge to the local context, it is important to identify the end user of the knowledge (CPGs).[3] The present study is targeting KT strategies to neurologists. Dissemination of the CPG should be tailored to the end user of the CPG. Dissemination

is commonly done in passive ways such as in academic journals and at conferences, which has not been found to be very effective.[4-6] More active dissemination of CPGs is needed and should include CPG-related products to facilitate the implementation into clinical practice. Such CPG-related products could include but are not limited to: summary brochures, training modules, patient-directed materials, quality indicators or performance metrics, and clinical decision support tools available at the point of care. Having access to the CPG and CPG-related products at the point of care (i.e. through the development of apps or tools incorporated into electronic medical records) would facilitate the use of CPGs clinically.[4]

Moreover, in the current digital era of social media, this mode of dissemination should not be discounted and should be leveraged to increase the dissemination of the CPG.[7] However, it is important that CPG dissemination be done by a reputable professional organization to maintain credibility, which is a determinant of CPG use.

If the CPG does not seem applicable to the local context in which it is being implemented, tools aimed at adapting the CPG should be used. The ADAPTE process is one such tool that adapts a CPG to the local context, while maintaining the integrity of the CPG.[8]

Intervention strategies aimed at adapting the CPG to the local context link to the following determinants of CPG use identified by our study: knowledge, credibility, applicability, resources, and target user.

Selecting & tailoring implementation interventions

The implementation interventions should be targeted to the identified barriers. In this case, the identified main determinants of CPG use among neurologists were credibility, knowledge, applicability, motivation, resources, and target users.

Nearly all KT strategies involve education as an intervention. As with dissemination, many forms of education are considered passive interventions and are frequently ineffective when used alone.[4-6] It is suggested that these methods be used in combination with other strategies suggested below, and that the education component be more interactive. Education intervention strategies link to the following determinant of CPG use among neurologists identified in our study: knowledge.

Persuasion, incentivisation, and enablement are suggested intervention functions linked to five of the identified determinants of CPG use among neurologists: knowledge, motivation, applicability, resources, and target users. Behaviour change interventions to address these functions include: reminders, outreach, audit and feedback, and patient-directed interventions.[9,10] Reminders (including outreach), and audit and feedback are some of the most effective CPG implementation interventions, and are recommended for other neurological conditions.[4,5,11] Reminders can be either electronic prompts, as in electronic medical record systems that alert physicians to a CPG if it applies to the management of a patient, or can be done more traditionally by re-dissemination of the CPG.[12] However, the frequency and appropriateness of the use of electronic reminders needs to be carefully considered as if they are too frequent or of little importance physicians may ignore them. Outreach, where an educator provides support to health

professionals in performing the care outlined in CPGs, can also act as a type of reminder.[12] Audit and feedback refers to the evaluation of a health professional's or group of professionals' performance, which is provided to them and may include suggested actions.[12] Reminders combined with audit and feedback is the most effective implementation strategy for CPGs.[4,5] Another intervention that is effective is patient-mediated intervention, especially when combined with other techniques.[4,5] Patient-mediated interventions use the patient, such as patient outcomes.[12] For example, the patient can provide the health professional with seizure diaries to illustrate the effect of an intervention on seizure frequency. Along this line, patients can also be used as the target of the reminders, although this intervention strategy has not been as well studied and has had varying results with regards to effectiveness.[6,13] These types of intervention link to the following determinants of CPG use among neurologists identified in our study: knowledge, motivation, applicability, resources, and target audience.

Modeling was also identified for five of the determinants of CPG use as an intervention function:[10] knowledge, credibility, motivation, applicability, and resources. The use of opinion leaders, identifiable as local leaders in CPG use, is a method of modeling CPG use.[12] The effectiveness of local opinion leaders varies considerably between studies and therefore its success as a behavior change intervention is unclear. Given the heterogeneity in the evidence, it is recommended that opinion leaders be used in conjunction with other behavior change interventions such as outreach or education.

Environmental restructuring, restriction, and coercion are other intervention functions that were mapped to the determinants of CPG use among neurologists. Specific to the

implementation of CPGs, environmental restructuring refers to changes in the physical environment (i.e. hospital) to encourage the use of CPGs.[10] An example of environmental restructuring is the introduction of prompts and reminders in electronic medical records. Restriction refers to limiting the opportunity to deviate from the recommended care outlined in the CPG.[10] An example of restriction could be limiting the ability of emergency physicians to order an MRI in inappropriate circumstances based on the recommended care in CPGs. Coercion is similar in that it would introduce a cost to ordering an inappropriate MRI.[10] These intervention functions could be addressed using reminders built into electronic medical records at the point of care, which has been discussed or by using a pay-per-performance model with penalty for inappropriate care.

<u>Evaluate knowledge use, evaluate outcomes & sustaining knowledge use</u>

The effectiveness of KT interventions will be only as strong as the level of knowledge used; therefore, before evaluating the outcome of the KT intervention, the degree of knowledge use must first be measured.[3]

Many of the principles used to evaluate any other intervention (i.e. a medical therapy) apply to evaluating KT interventions. The accepted gold standard for evaluating an intervention is a RCT. While RCTs are less frequently undertaken in KT they have been utilized to evaluate intervention strategies.[4,15] A RCT is not always a feasible design given the pragmatic nature of KT studies. Other evaluation methods often used in KT include: before-and-after designs, step-wedge quasi-experimental designs and

interrupted time series design.[4] The type of design chosen will depend on the exact research question, the intervention, the clinical setting and resources available.

## References


1. Sauro K, Wiebe S, Dunkley C, et al. Current State of International Epilepsy Guidelines. Epilepsia. 2016;57:13-23.
2. Browman GP, Newman TE, Mohide EA, et al. Progress of clinical oncology guidelines development using the Practice Guidelines Development Cycle: the role of practitioner feedback. J Clin Oncol. 1998;16:1226-31.
3. Graham ID, Logan J, Harrison MB, et al. Lost in knowledge translation: time for a map? J Contin Educ Health Prof. 2006;26:13-24.
4. Grimshaw JM, Thomas RE, MacLennan G, et al. Effectiveness and efficiency of guideline dissemination and implementation strategies. Health Technol Assess. 2004;8:iii-iv, 1-72.
5. Oxman AD, Thomson MA, Davis DA, et al. No magic bullets: a systematic review of 102 trials of interventions to improve professional practice. CMAJ. 1995;153:1423-31.
6. Wensing M, van der Weijden T, Grol R. Implementing guidelines and innovations in general practice: which interventions are effective? Br J Gen Pract. 1998;48:991-7.
7. Van Noorden R. Online collaboration: Scientists and the social network. Nature. 2014;512:126-9.
8. Harrison MB, Legare F, Graham ID, et al. Adapting clinical practice guidelines to local context and assessing barriers to their use. CMAJ. 2010;182:E78-84.
9. Michie S, Johnston M, Abraham C, et al. Making psychological theory useful for implementing evidence based practice: a consensus approach. Qual Saf Health Care. 2005;14:26-33.
10. Michie S, van Stralen MM, West R. The behaviour change wheel: A new method for characterising and designing behaviour change interventions. Implementation Science. 2011;6:42.
11. Schunemann HJ, Cook D, Grimshaw J, et al. Antithrombotic and thrombolytic therapy: from evidence to application: the Seventh ACCP Conference on Antithrombotic and Thrombolytic Therapy. Chest. 2004;126:688S-96S.
12. EPOC Taxonomy 2015. Available from: https://epoc.cochrane.org/epoc-taxonomy.
13. Burack RC, Gimotty PA, George J, et al. The effect of patient and physician reminders on use of screening mammography in a health maintenance organization. Results of a randomized controlled trial. Cancer. 1996;78:1708-21.
14. Sauro K, Wiebe S, Holroyd-Leduc J, et al. Determinants of Clinical Practice Guideline Use Among Neurologists: A mixed-methods approach. 2016.
15. Davis J, Roberts R, Davidson D, et al. Implementation strategies for a Scottish natioal epilepsy guideline in primary care: results of the Tayside Implementation of Guidelines in Epilepsy Randomized (TIGER) trial. Epilepsia. 2004;45:28-34.
