# Supplementary figures and images for: Knowledge translation of clinical practice guidelines among neurologists: A mixed-methods study
Source: PLoS One. 2018 Oct 10;13(10):e0205280. doi: 10.1371/journal.pone.0205280 (PMC6179253; doi:10.1371/journal.pone.0205280)

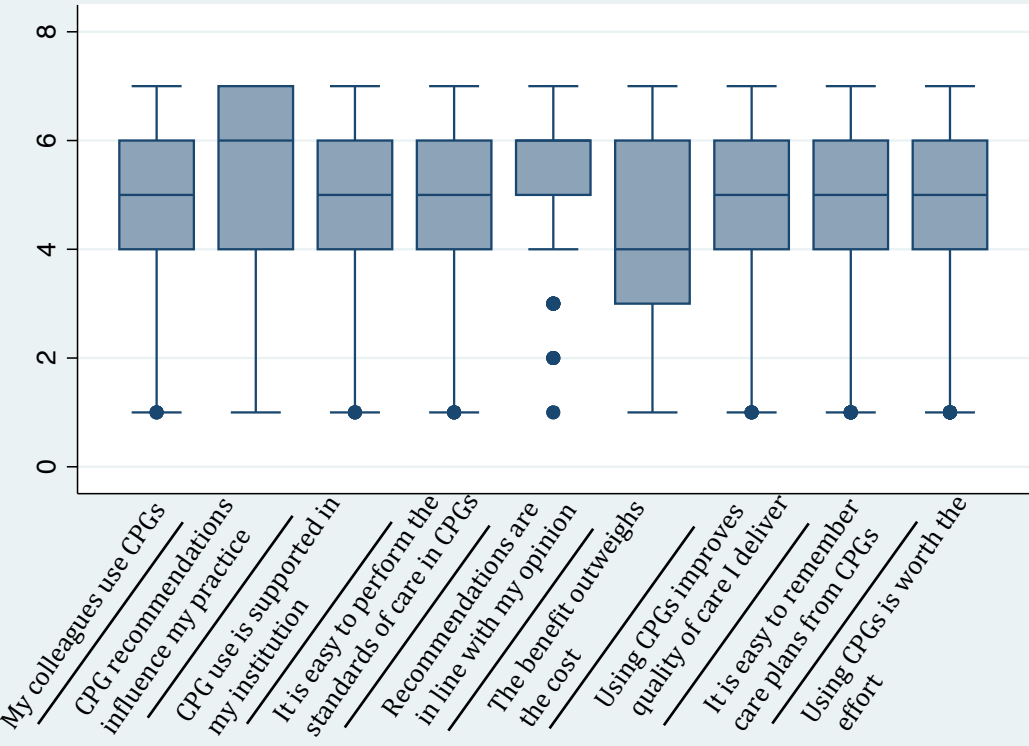

Supplement: S1 Fig — (PDF) [file pone.0205280.s007.pdf]

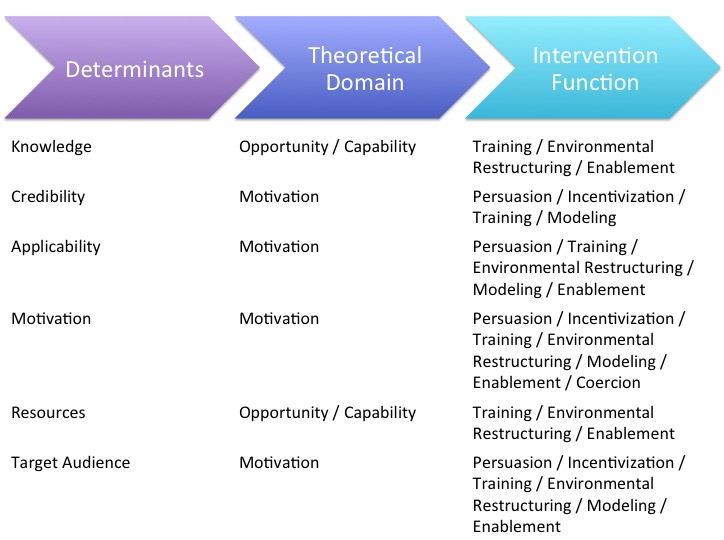

Supplement: S2 Fig — (JPG) [file pone.0205280.s008.jpg]
