# Supplementary material for: Knowledge translation of clinical practice guidelines among neurologists: A mixed-methods study
Source: PLoS One. 2018 Oct 10;13(10):e0205280. doi: 10.1371/journal.pone.0205280 (PMC6179253; doi:10.1371/journal.pone.0205280)
Supplement: S1 Table — (PDF) [file pone.0205280.s009.pdf]

**Logistic regression using the dichotomous CPG use question "Do you use clinical practice guidelines in your practice?"**				
**Variable**	**OR**	**Lower 95% CI**	**Upper 95% CI**	**p-value**
Urban vs. rural	0.67	0.33	1.33	0.25
Sex	0.40	0.20	0.82	0.01
Academic affiliation	2.95	1.29	6.78	0.01
General vs. subspecialty	0.98	0.69	1.39	0.33
**Linear regression using the continuous CPG use question**				
	**Coefficient**	**Lower 95% CI**	**Upper 95% CI**	**p-value**
Urban vs. rural	-0.24	-0.61	0.13	0.21
Sex	-0.09	-0.53	0.35	0.68
Academic affiliation	0.38	-0.26	1.03	0.24
General vs. subspecialty	-0.05	-0.29	0.20	0.71

Abbreviations: **CPG**=clinical practice guidelines, **95% CI**=95% confidence interval, **OR**=odds ratio
