# Supplementary material for: Knowledge translation of clinical practice guidelines among neurologists: A mixed-methods study
Source: PLoS One. 2018 Oct 10;13(10):e0205280. doi: 10.1371/journal.pone.0205280 (PMC6179253; doi:10.1371/journal.pone.0205280)
Supplement: S2 Table — (PDF) [file pone.0205280.s010.pdf]

S2 Table. CPG use and determinants of CPG use for profiles of responders and non-responders

	All* n (% Yes)	Responder n=292 (93.3%)	Non-responder n=21 (6.7%)	p-value (X^2)
Do you use CPGs in your practice?	242 (81.0)	234 (81.0)	8 (61.5)	0.086
**Barrier**				
There are incentives to follow CPGs in my practice	29 (9.7)	27 (9.4)	2 (16.7)	0.405
Lack of knowledge about CPGs is a barrier to their use in my practice	121 (41.2)	116 (41.1)	5 (41.7)	0.971
Time constraints are a barrier to the use of CPGs in my practice	115 (38.7)	109 (38.3)	6 (50.0)	0.413
The applicability of CPGs to my clinical setting is a barrier to their use	101 (34.8)	99 (35.5)	2 (18.2)	0.237
I do not have the skills to perform the standards of care recommended in most CPGs	12 (4.1)	11 (3.9)	1 (9.1)	0.394
I do not have the resources to perform the standards of care recommended in most CPGs	40 (13.8)	38 (13.6)	2 (20.0)	0.566
	**All**	**Responder**	**Non-responder**	
	**Median (IQR)**	**Median (IQR)**	**Median (IQR)**	
I use CPGs in my clinical practice	5 (3)	5 (2)	5.5 (2)	0.833
**Determinants**				
My colleagues use CPGs in their clinical practice	5 (2)	5 (2)	5 (3)	0.668
CPG recommendations influence my clinical practice	6 (3)	5 (3)	6 (2)	0.413
The use of CPGs is supported in my institution	5 (2)	5 (2)	6 (2)	0.246
It is easy to perform standards of care outlined in CPGs	5 (2)	5 (2)	5 (2)	0.996
Recommendations are often in line with my professional opinion	6 (1)	6 (1)	6 (1)	0.250
The benefit of using CPGs outweighs the costs	4 (3)	4 (3)	4.5 (5)	0.991

Following CPGs improves the quality of care I deliver	5 (2)	5 (2)	6 (3)	0.593
It is easy to remember the care plan outlined in CPGs when I see patients	5 (2)	5 (2)	6 (2)	0.051
Using CPGs in my practice is worth the effort	5 (2)	5 (2)	6 (2)	0.212

Abbreviations: **CPG**=clinical practice guideline; **IQR**=interquartile range (q75-q25); **n**=number of participants; **p-value**=p-value of comparisons between responders and non-responders
